# Research on fractional-order memory system signals based on Loop-By-Loop Progressive Iterative Method

**DOI:** 10.1038/s41598-024-75537-4

**Published:** 2024-10-18

**Authors:** Li Xu, Chuan Huang, Guo Huang, Duyi He

**Affiliations:** 1 School of Electronic Information and Artificial Intelligence, Leshan Normal University, Leshan, China; 2 Leshan Normal University Library, Leshan, China

**Keywords:** Fractional-order memory systems output signals, Fractance, Flux-controlled fractional-order memory systems, Loop-By-Loop Progressive Iterative Method, Engineering, Physics

## Abstract

This article abandons the traditional Laplace transform and proposes a new method for studying fractional-order circuits, which is the Loop-By-Loop Progressive Iterative Method(LPIM). Firstly, in order to demonstrate the correctness of LPIM, the fractance circuit, which is a relatively mature and simple form in fractional-order circuits, was chosen as the research object. The output signals of fractance circuit were studied for the first time using Laplace transform and LPIM, respectively. The results showed that the conclusions obtained by LPIM were completely consistent with those obtained by Laplace transform method and existing theories, thus verifying the correctness of LPIM. Then, a brand new Fractional-Order Memory Systems (FMS) model is constructed, and based on this model, LPIM is used for the first time to simulate the output signal of Flux-Controlled Fractional-Order Memory Systems (FFMS) that has not been studied so far. The results show that when a sine signal is used as the excitation signal, the output signal of the FFMS intersects at two points, and the output signal is modulated by the frequency of the excitation signal. Finally, combining existing theories, predict the output commonalities of FMS.

## Introduction

At the beginning of the 21st century, fractional calculus has received extensive attention, and almost all calculus fields are studying fractional calculus. So far, fractional calculus has been applied to many scientific fields, such as fractional viscoelastic mechanics, fractional diffusion process, fractional image processing, fractional signal processing, fractional control, fractional neural networks, fractal dynamic systems, fractional circuits and systems, etc^1–6^. Because fractional calculus has the characteristics of nonlocality, weak singularity and long-term memory, physical scientists and engineering technicians regard it as a new mathematical method. Fractional order systems are known as systems of the 21st century^7^. Fractance is the abbreviation of fractional impedance, which is the name of fractional components in electromagnetism, electrical electronics, signal and information processing, control theory and other fields. With fractor elements, fractional order calculus circuits and fractional order control can be realized, and various fractional order processes and phenomena in nature, chaotic processes and phenomena in nonlinear systems and fractal processes and phenomena can be simulated and studied on the basis of electronic information theory, technology and methods. So as to observe and analyze various materials, biological tissues, organs, complex real system behaviors, as well as a large number of fractional calculus phenomena in physics, biology, chemistry, medicine, control and other fields.

As is well known, Leon Chua considered multiple aspects such as logical compatibility, axiomatic completeness, and formal symmetry, and proposed that there should be fourth basic circuit component—memristor^8–13^. Memristor is a nonlinear resistor with memory function, which can remember the amount of charge and magnetic flux flowing through it. It can change the resistance value by controlling the changes in current or voltage, and this change can continue to be maintained in the event of a power outage, making memristor a natural non-volatility storage device^14–17^. Memristors can be used in new types of memory^18–23^, artificial intelligence computers^24,25^, artificial neural networks^26–28^, secure communication, chaotic oscillation circuits^29,30^, image processing^31,32^, etc. The memory characteristics of memristors will have extremely profound impacts on computer science, biotechnology, neural networks, electronic engineering, communication engineering, and more.

Fractance is composed of three basic components of a circuit: capacitance and resistance constitute capacitive fractance, and inductance and resistance constitute inductive fractance. As the fourth missing basic component, memristors can also be used to construct fractional order circuits^33–44^.

In 2016, Pu Yifei and others first constructed a 1/2-order net-grid-type Fractional-Order Memory System (FMS) based on Chua’s axiomatic element system and fractional order calculus theory^45^. And the impedance formula of any order FMS was derived as follows:

1 $$\:{\text{F}\text{M}}_{-\text{p}}^{\text{c}}={\text{m}}^{1-\text{p}}{\text{C}}^{-\text{p}}{\text{s}}^{-\text{p}} \quad 0\le\:p\le\:1,$$

2 $$\:{\text{F}\text{M}}_{\text{p}}^{\text{l}}={\text{m}}^{1-\text{p}}{\text{L}}^{\text{p}}{\text{s}}^{\text{p}} \quad 0\le\:\text{p}\le\:1,$$

where m represent the impedances of memristors; C and L denote capacitance and inductance, respectively; p is the fractional order, s is the Laplace variable, and $\:{\text{F}\text{M}}_{-\text{p}}^{\text{c}}$represents the impedance of any order capacitive FMS, $\:{\text{F}\text{M}}_{-\text{p}}^{\text{c}}$represents the impedance of any order of inductive FMS. In 2018, Yifei et al.^46^ simulated and implemented the first internationally recognized FMS in the laboratory, and conducted some research on the characteristics of FMS.

For the research of FMS signals, after obtaining the general impedance formulas (1) and (2), it becomes very difficult to carry out further research. This is mainly because of the particularity of memristor whose resistance values change with the change of current, which makes Laplace transform more complex and difficult to calculate. In addition, the introduction of fractional order makes it almost impossible to calculate the inverse Laplace transform of Eqs. (1) and (2). Memristors are a type of passive two terminal components, divided into two types: charge-controlled memristors and flux-controlled memristors. Currently, all research on FMS uses charge-controlled memristors because flux-controlled memristors are a function of magnetic flux, which depends on the voltage in the circuit, making it almost impossible to calculate using traditional Laplace transforms. Therefore, the understanding of FMS is very limited; especially the properties of Flux-Controlled Fractional-Order Memory Systems (FFMS) are unknown.

The theory and application fields of circuits and systems are undergoing an unprecedented transformation. Chua’s axiomatic component system theory and the fourth component—memristors, chaotic circuits, fractional-order circuits and systems are the foundation of this transformation. And FMS combine memristors with fractional-order circuits. However, the research on FMS has not been further advanced, and the fundamental reason is that it is difficult to solve. Scholars have been committed to solving similar complex systems, such as the Spectral Element Method (SEM), which can be used to solve certain complex electromagnetic systems due to its accuracy and low computational cost^47–49^. This article also aims to solve the FMS that traditional methods cannot solve well, and therefore proposes a new research method for fractional-order circuits. This method abandons the traditional Laplace transform and gradually solves the circuit loop by loop based on Kirchhoff’s voltage and current theorems, starting from the essence of the circuit. Therefore, it is called loop-by-loop progressive iteration method, abbreviated as LPIM. LPIM does not require any approximation in the solving process and can solve any form of FMS, making it possible to simulate the properties of any order FMS constructed by any form of memristor, and thus summarize the commonalities of FMS. This will undoubtedly promote the application research of FMS. FMS has enormous application prospects, among which its complex dynamic behavior makes it have great potential in chaotic oscillation circuits. However, its solving problems have always hindered its progress. The proposal of LPIM is expected to break through this bottleneck and open up a new situation in chaotic oscillation circuits research, which will also be our future research direction.

The remainder of this paper is organized as follow: To demonstrate the rationality of LPIM, firstly, based on the Laplace transform, the Oldham fractal chain fractance circuit output signals were simulated for the first time through operator approximation. Then, the same circuit properties were studied using the LPIM. The conclusions obtained by the two methods are completely consistent, which preliminarily proves the rationality of LPIM. So far, almost all of the FMS theory are based on net-grid-type structure, and this paper constructs a new Oldham fractal chain FMS. Then, the second-order nonlinear flux-controlled memristor is applied to this FMS, and a FFMS is constructed. In this case, the traditional Laplace transform is completely unable to solve the problem. Based on LPIM, the general differential equation system of FFMS was derived, and the differential equation system was solved using MATLAB, thus simulating the output signals of FFMS for the first time.

## LPIM and its validation

In order to verify the rationality of LPIM, the famous Oldham fractal chain structure circuit was taken as the research object. Based on the traditional Laplace algorithm, the output of the Oldham fractal chain fractance circuit was simulated for the first time through mathematical substitution; Then use the LPIM to study the same properties.

The Oldham fractal chain fractance circuit is a famous fractance approximation circuit, and its circuit structure is shown in the following figure:

**Fig. 1:**
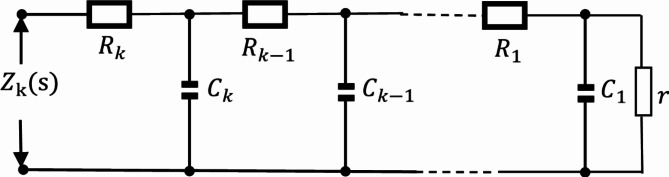
k-level Oldham chain fractance.

The Oldham fractal chain fractance circuit in Fig. 1 exhibits half order capacitance characteristics at low frequencies. Its impedance is:

3 $${{\text{Z}}_O}{\text{=}}\sqrt {\frac{R}{{Cs}}} ,$$

where R represent the resistance, and $\:{Z}_{0}$ represents the impedance of the fractance circuit.

### Research on the output signals of Oldham fractal chain fractance circuit based on Laplace transform

Let input voltage $V(t){\text{=}}{v_{\hbox{max} }}\sin (\omega t)$, and according to Laplace transform, obtain:

4 $$V(s){\text{=}}\frac{{{v_{\hbox{max} }}\omega }}{{{s^2}+{\omega ^2}}},$$

The the Laplace transform of the current in the circuit is:

5 $$I(s)=V/{Z_0}=\frac{{{v_{\hbox{max} }}\omega }}{{({s^2}+{\omega ^2}){R^{1/2}}{C^{ - 1/2}}{s^{ - 1/2}}}}.$$

If we want to study the output signal of the circuit, we need to perform an inverse Laplace transform on Eq. (5), that is

6 $$I(t)={L^{ - 1}}[I(s)]={L^{ - 1}}\left[\frac{{{v_{\hbox{max} }}\omega }}{{{R^{1/2}}{C^{ - 1/2}}{s^{ - 1/2}}({s^2}+{\omega ^2})}}\right],$$

in the Eq. (6), there is a fractional order operator${s^{{\text{-}}1/2}}$, so the inverse Laplace transform cannot be performed. Therefore, the fractional order operator ${s^{{\text{-}}1/2}}$ is approximated as follows:

7 $${s^{ - 1/2}}=\frac{{{s^5}+55{s^4}+330s{}^{3}+462{s^2}+165s+11}}{{11{s^5}+165{s^4}+462{s^3}+330{s^2}+55s+1}},$$

by substituting Eq. (7) into Eq. (6) and solving it using MATLAB, the output signals of the circuit can be obtained.

Let $R{\text{=}}4\Omega$, $C=0.5\,F,$ ${v_{\text{max}}}=2\,V$; Simulate the V-I diagram of the ideal fractance circuit output signal at different voltage frequencies:

**Fig. 2:**
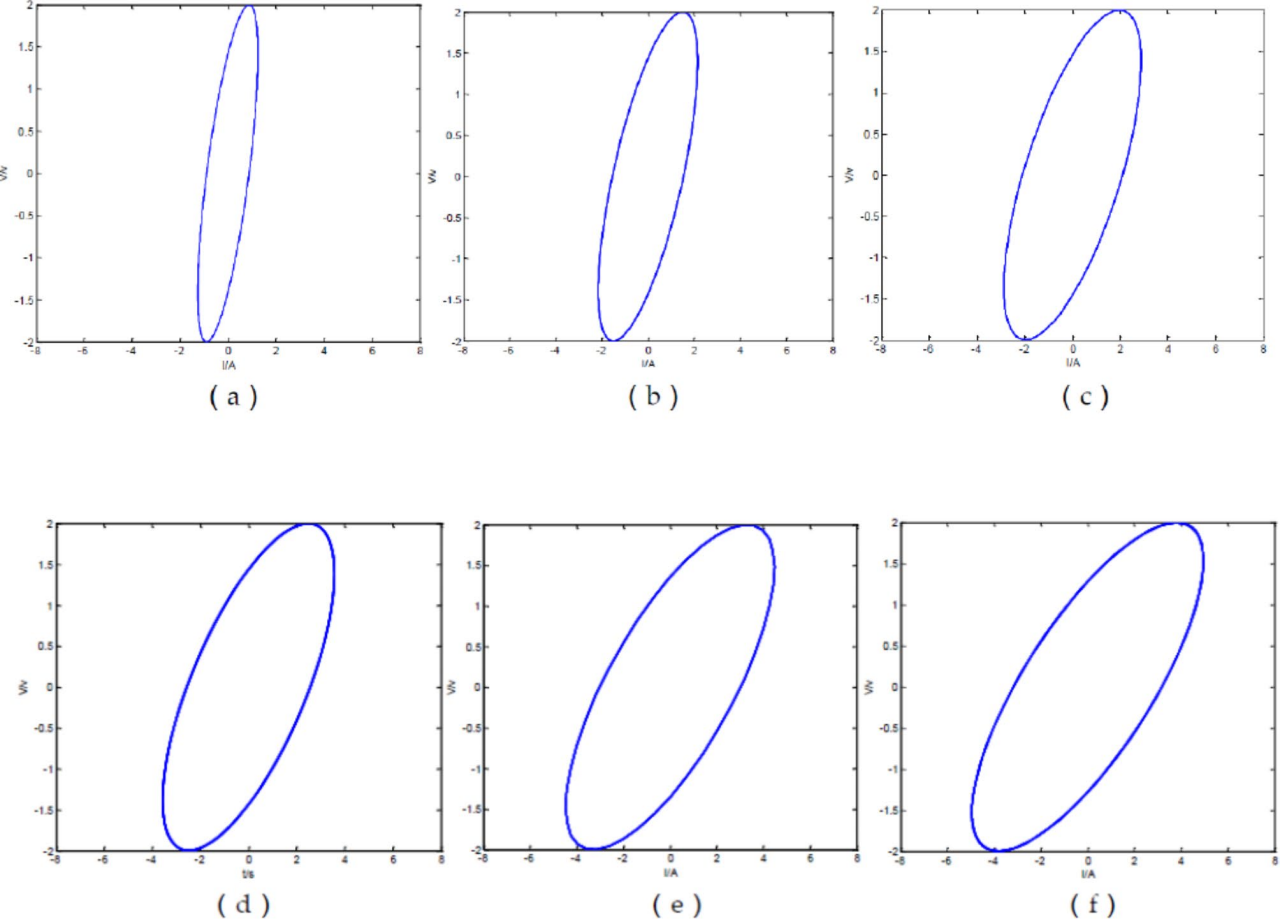
V-I diagram of ideal fractance circuit with different driving voltage frequencies. (**a**) V-I diagram of the output signals when$\omega {\text{=1}}\pi$; (**b**) V-I diagram of the output signals when $\omega {\text{=3}}\pi$; (**c**) V-I diagram of the output signals when $\omega {\text{=5}}\pi$; (**d**) V-I diagram of the output signals when $\omega {\text{=7}}\pi$; (**e**) V-I diagram of the output signals when $\omega {\text{=10}}\pi$; (**f**) V-I diagram of the output signals when$\omega {\text{=12}}\pi$

From the Fig. 2, we can see that the V-I diagram of the output signal is a regular ellipse. The area of the ellipse increases with the increase of voltage frequency and the elliptical position presents a state of gradually clockwise change with frequency change.

The graph of the total current of the output signal over time at different driving voltage frequencies is as follows.

**Fig. 3:**
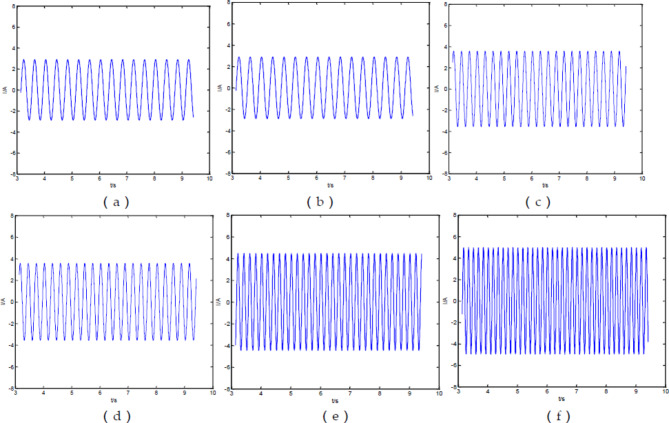
Total current versus time of ideal fractance circuit with different driving voltage frequencies. (**a**) I-t diagram of the output signals when$\omega {\text{=1}}\pi$; (**b**) I-t diagram of the output signals when$\omega {\text{=3}}\pi$; (**c**) I-t diagram of the output signals when$\omega {\text{=5}}\pi$; (**d**) I-t diagram of the output signals when $\omega {\text{=7}}\pi$; (**e**) I-t diagram of the output signals when$\omega {\text{=10}}\pi$; (**f**) I-t diagram of the output signals when$\omega {\text{=12}}\pi$

From the Fig. 3, we can see that as the voltage frequency increases, the current frequency of the output signal also increases, and the current amplitude gradually increases too.

### Solving 1/2 order Oldham fractal chain fractance circuits using LPIM

From the above solution, it can be seen that the introduction of fractional order results in greater difficulty in solving. This article proposes a new method for solving fractional order circuits. This method starts from the essence of the circuit and is based on the Kirchhoff voltage and current theorem to gradually solve the circuit loop by loop. Therefore, it is named as loop-by-loop progressive iteration method, abbreviated as LPIM. In order to verify the rationality of LPIM, this method was applied to solve the Oldham fractal chain fractance circuit, and the output signal of the circuit was obtained.

As mentioned above, the structure diagram of the Oldham fractal chain fractance circuit is as follows:


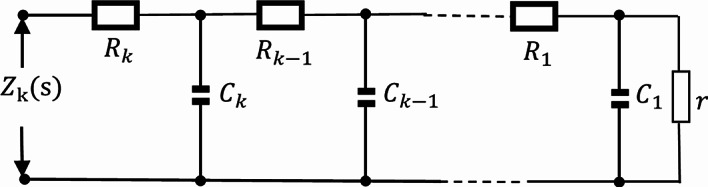


A primitive with a similar structure:
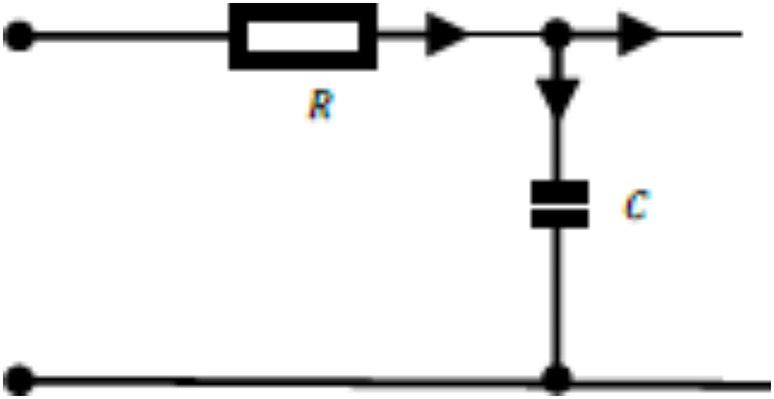


is called a loop. Split the circuit, when there is only one loop in the circuit:

**Fig. 4:**
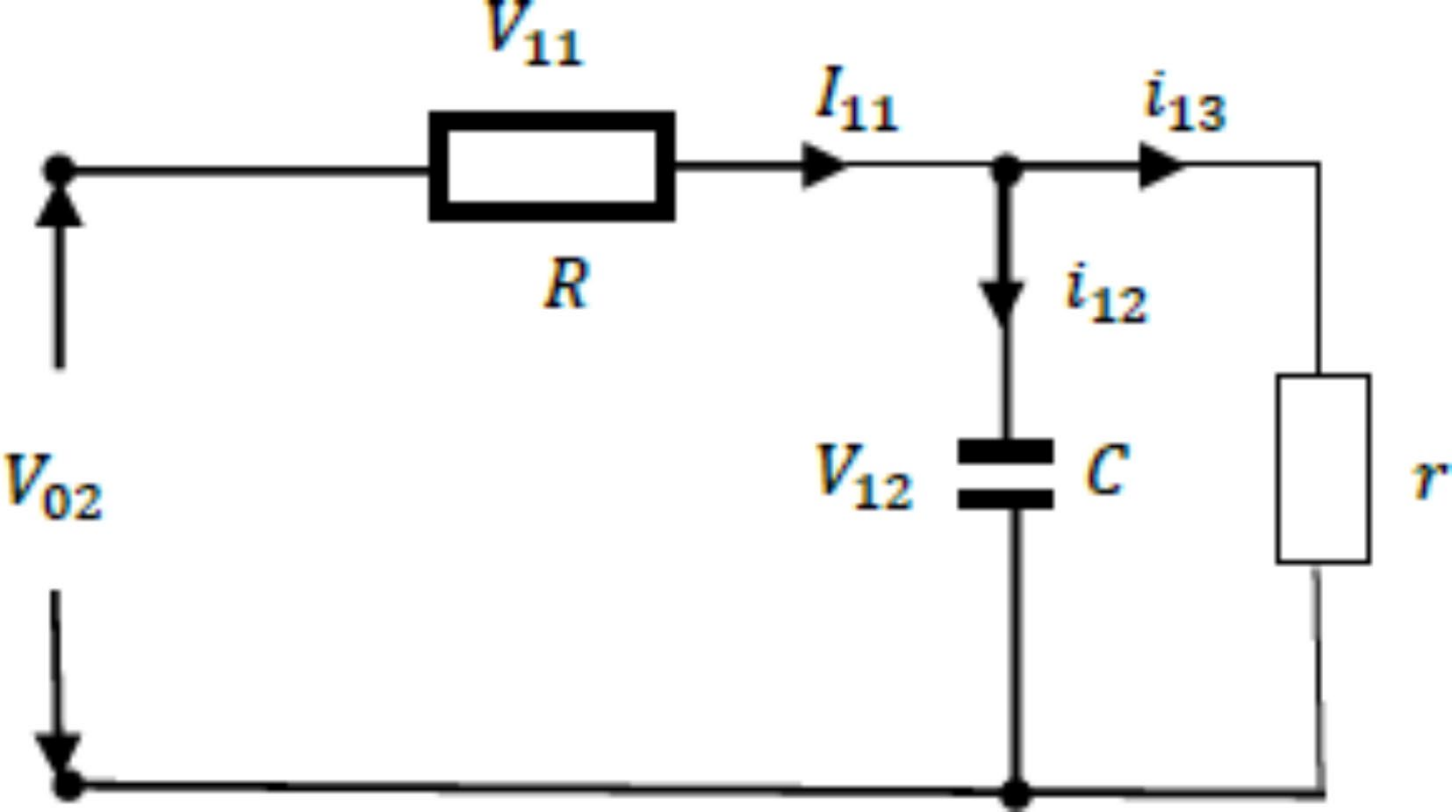
The Oldham chain fractance circuit with only one loop.

As shown in the Fig. 4, set resistance as R, capacitance as C, external load resistance as r, and express the total voltage as $\:{\text{V}}_{02}$, the voltage between both ends of the resistor is $\:{\text{V}}_{11}$. The voltage at both ends of the capacitor is $\:{\text{V}}_{12}$. The main circuit current is $\:{\text{I}}_{11}$. The two branch currents are $\:{\text{i}}_{12}$ and $\:{\text{i}}_{13}$ respectively. According to Kirchhoff’s voltage and current theorem, it can be concluded that:

8 $${V_{02}}={V_{11}}+{V_{12}},$$

9 $${I_{11}}={i_{12}}+{i_{13}}.$$

According to Ohm’s law:

10 $$\frac{{{V_{11}}}}{R}={I_{11}},$$

11 $$\frac{{{V_{12}}}}{r}={i_{13}}.$$

According to the definition of capacitance:

12 $$\frac{{d{V_{12}}}}{{dt}}=\frac{{{i_{12}}}}{C}.$$

Substituting (8) (10) (11) (12) into (9) yields:

13 $$\frac{{d{V_{12}}}}{{dt}}=\frac{{{V_{02}}}}{{CR}} - \left( {\frac{1}{{CR}}+\frac{1}{{Cr}}} \right){V_{12}}.$$

Equation (13) is a solvable first order differential equation. When there are two loops in the circuit, the circuit structure is:

**Fig. 5:**
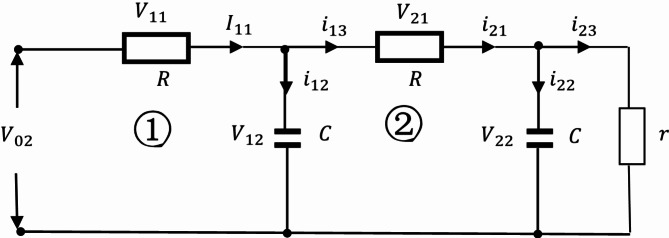
The Oldham chain fractance circuit with two loops.

The representation of each physical quantity is shown in Fig. 5. The capacitor voltage of the first loop is $\:{\text{V}}_{12}$ and that of the second loop is $\:{\text{V}}_{22}$; The resistance voltage of the first loop is $\:{\text{V}}_{11}$ and that of the second loop is $\:{\text{V}}_{21}$; The branch currents of the first loop are ${i_{12}}$ and ${i_{13}}$ respectively and that of the second loop is ${i_{21}}$, ${i_{22}}$, ${i_{23}}$. If P represents all physical quantities, i and j are natural numbers, and all of the above physical quantities are represented as $\:{\text{P}}_{\text{i}\text{j}}$ where i is the number of loops and j is the corresponding physical quantity ordinal. Ordinal numbers ① and ② represent the first and second loops, respectively.

From loop ①, it can be concluded that:

14 $$\left\{ {\begin{array}{*{20}{l}} {{V_{02}}={V_{11}}+{V_{12}}} \\ {{I_{11}}={i_{12}}+{i_{13}}} \\ {\frac{{{V_{11}}}}{R}={I_{11}}} \\ {\frac{{d{V_{12}}}}{{dt}}=\frac{{{i_{12}}}}{C}} \\ {{i_{13}}={i_{21}}} \end{array}} \right.,$$

After substituting Eq. (14), we obtain:

15 $$\frac{{d{V_{12}}}}{{dt}}=\frac{{{V_{02}} - {V_{12}}}}{{RC}} - \frac{{{i_{21}}}}{C}.$$

From loop ②, it can be concluded that:

16 $$\left\{ {\begin{array}{*{20}{l}} {{V_{12}}={V_{21}}+{V_{22}}} \\ {{i_{21}}={i_{22}}+{i_{23}}} \\ {\frac{{{V_{21}}}}{R}={i_{21}}} \\ {\frac{{d{V_{12}}}}{{dt}}=\frac{{{i_{22}}}}{C}} \\ {\frac{{{V_{22}}}}{r}={i_{23}}} \end{array}} \right. .$$

According to Eq. (16), it can be concluded that:

17 $${i_{21}}{\text{=}}\frac{{{V_{21}}}}{R}=\frac{{{V_{12}} - {V_{22}}}}{R},$$

Substituting Eq. (17) into Eq. (15) yields:

18 $$\frac{{d{V_{22}}}}{{dt}}=\frac{{{V_{12}}}}{{CR}} - \left( {\frac{1}{{CR}}+\frac{1}{{Cr}}} \right){V_{22}}.$$

After changing Eq. (16), it can be concluded that:

19 $$\frac{{d{V_{22}}}}{{dt}}=\frac{{{V_{12}}}}{{CR}} - \left( {\frac{1}{{CR}}+\frac{1}{{Cr}}} \right){V_{22}}.$$

So, we can obtain the equation system:

20 $$\left\{ {\begin{array}{*{20}{l}} {\frac{{d{V_{12}}}}{{dt}}=\frac{{{V_{02}}+{V_{22}} - 2{V_{12}}}}{{CR}}} \\ {\frac{{d{V_{22}}}}{{dt}}=\frac{{{V_{12}}}}{{CR}} - \left( {\frac{1}{{CR}}+\frac{1}{{Cr}}} \right){V_{22}}} \end{array}} \right.,$$

Equation (20) is a solvable system of first order differential equations.

When there are three loops in the circuit, the circuit structure is:

**Fig. 6:**
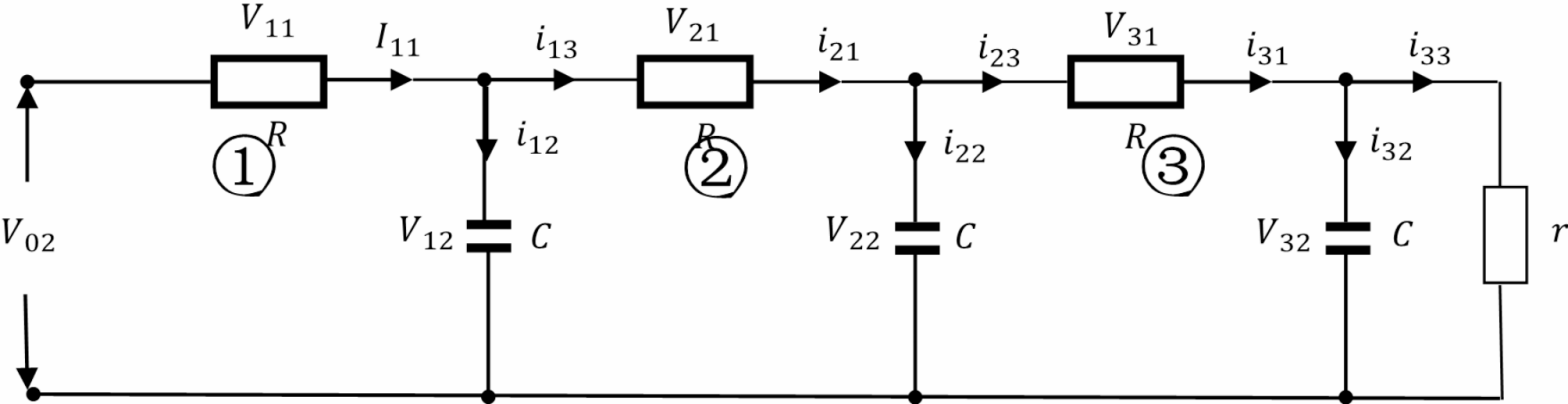
The Oldham chain fractance circuit with three loops.

The representation of each physical quantity in Fig. 6 is the same as in Fig. 5. Similarly, we obtain the differential equation from loop ① as follows:

21 $$\frac{{d{V_{12}}}}{{dt}}=\frac{{{V_{02}}+{V_{22}} - 2{V_{12}}}}{{CR}}.$$

Obtain the differential equation from loop ②:

22 $$\frac{{d{V_{22}}}}{{dt}}=\frac{{{V_{12}}+{V_{32}} - 2{V_{22}}}}{{CR}}.$$

The differential equation of loop ③ is:

23 $$\frac{{d{V_{32}}}}{{dt}}=\frac{{{V_{22}}}}{{CR}} - \left( {\frac{1}{{CR}}+\frac{1}{{Cr}}} \right){V_{32}}.$$

By analogy, when the circuit has n loops:

**Fig. 7:**
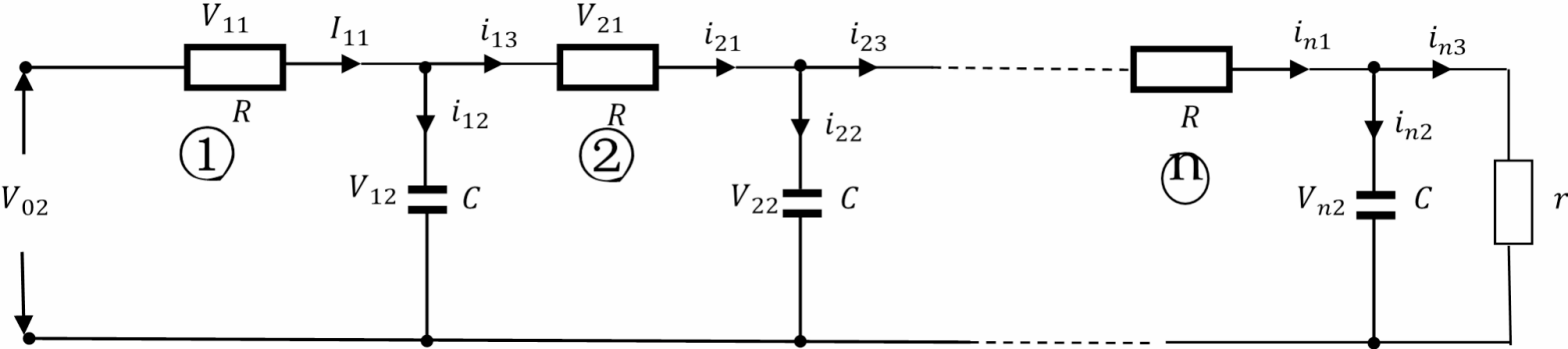
The Oldham chain fractance circuit with n loops.

Figure 7 shows the Oldham chain fractance circuit structure with n loops, and its differential equations system is:

24 $$\left\{ {\begin{array}{*{20}{l}} {\frac{{d{V_{k2}}}}{{dt}}=\frac{{{V_{(k - 1)2}}+{V_{(k+1)2}} - 2{V_{k2}}}}{{CR}} \quad k=1,2,3, \ldots, n - 1} \\ {\frac{{d{V_{k2}}}}{{dt}}=\frac{{{V_{(k - 1)2}}}}{{CR}} - \left( {\frac{1}{{CR}}+\frac{1}{{Cr}}} \right){V_{k2}}, \quad k=n} \end{array}} \right.$$

Equation (24) is a solvable first-order differential equation system, and the number of differential equations depends on the number of loop n. By using MATLAB, the differential equation system can be solved to study the output signals. When the input voltage$\:{\text{V}}_{1}={\text{V}}_{\text{m}\text{a}\text{x}}\text{s}\text{i}\text{n}\left({\upomega\:}\text{t}\right)$, $\:\:{\text{V}}_{\text{m}\text{a}\text{x}}=3\text{V}$, $\:{\upomega\:}={\uppi\:}$, $\:\text{R}=30{\Omega\:}$, external load $\:\text{r}=2\times\:{10}^{5}{\Omega\:}$, $\:\text{C}=0.3{\upmu}\text{F}$, and the number of circuit loops is different, Eq. (24) can be solved using MATLAB, and the V-I diagram of the output signal can be simulated as follows:

**Fig. 8:**
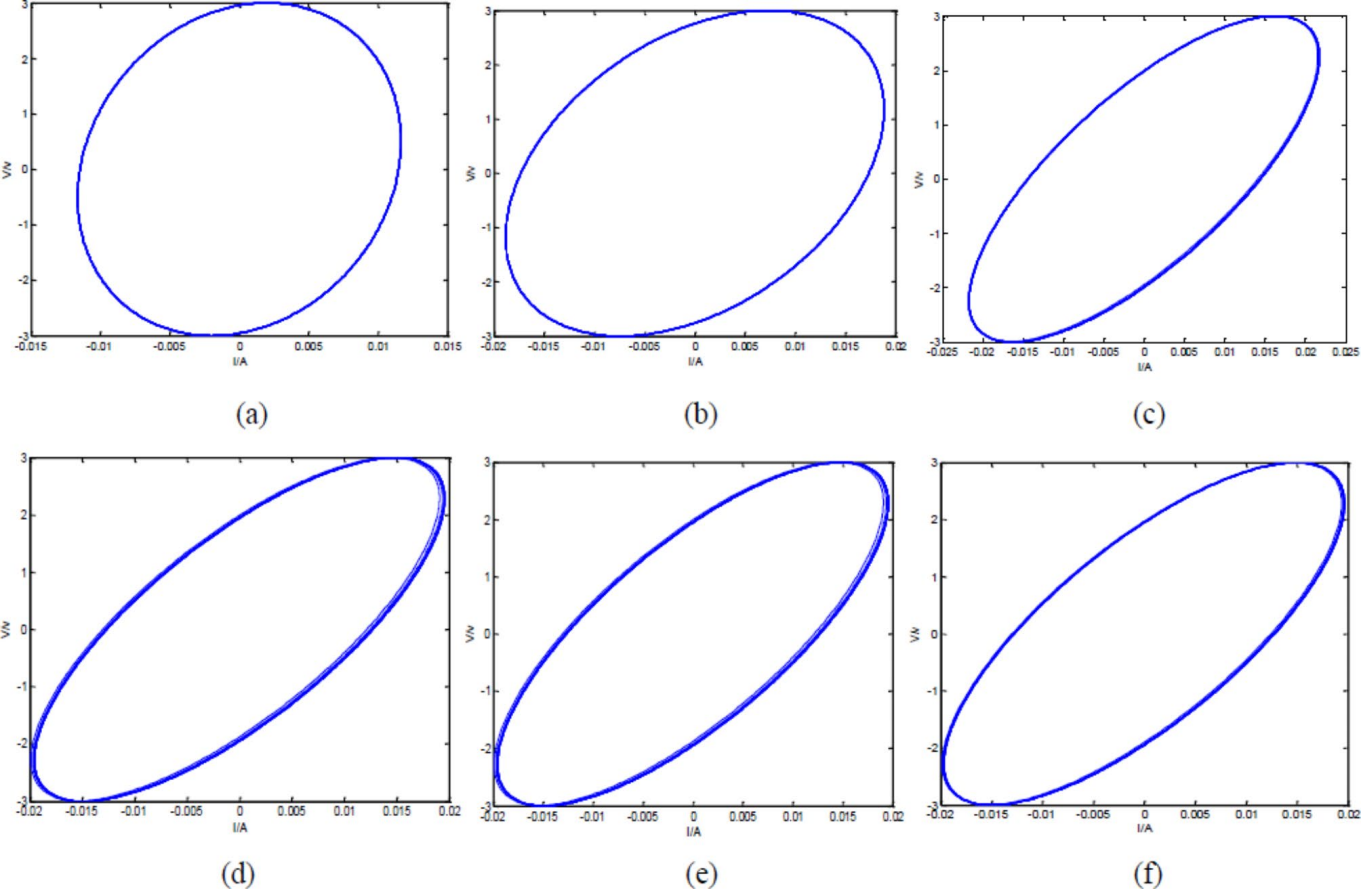
V-I diagram of Oldham chain fractance circuit with different loop numbers. (**a**) V-I diagram of the output signals with three loops; (**b**) V-I diagram of the output signals with five loops; (**c**) V-I diagram of the output signals with ten loops; (**d**) V-I diagram of the output signals with twenty loops; (**e**) V-I diagram of the output signals with thirty loops; (**f**) V-I diagram of the output signals with forty loops.

From Fig. 8, it can be observed that when the number of loops in the circuit is three, the V-I diagram of the output signal is similar to a circle, and the output of the circuit is different from the ideal fractance. When the number of circuit loops is five, the V-I diagram becomes elliptical and has the characteristic of ideal fractance. As the number of loops increases, the fractance characteristics of the circuit become more obvious, and the circuit characteristics at ten loops are not significantly different from the final V-I diagram. The V-I diagrams of the twenty loops, thirty loops, and forty loops circuits have not changed much. The above conclusion is completely consistent with existing theories.

When the circuit structure is forty loops, the driving voltage frequency changes, and other parameters remain unchanged, the V-I diagram of the output signal is as follows.

**Fig. 9:**
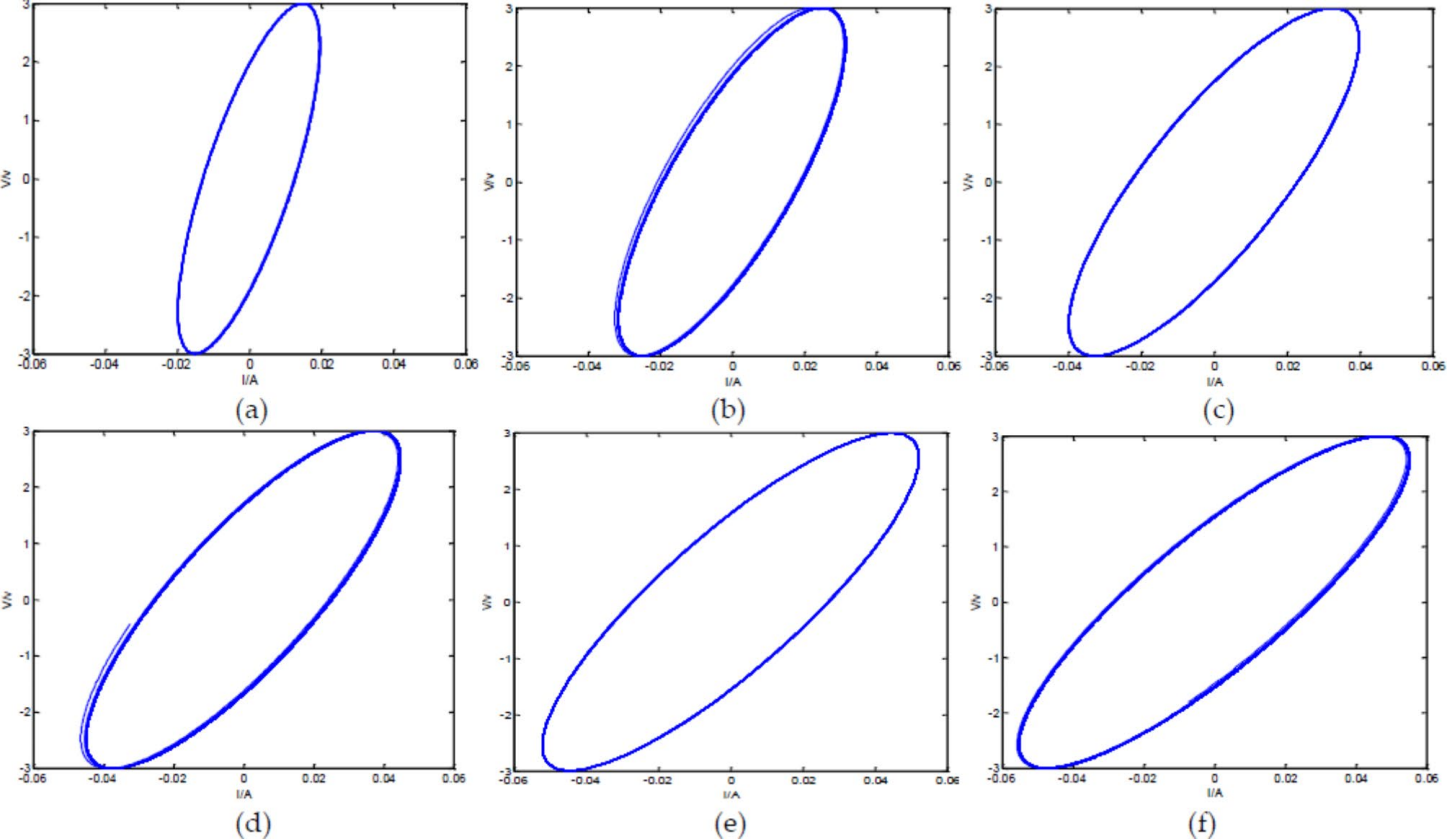
V-I diagram of Oldham chain fractance circuit with forty loops at different frequencies. (**a**) V-I diagram of the output signals when ω = 1π; (**b**) V-I diagram of the output signals when ω = 3π; (**c**) V-I diagram of the output signals when ω = 5π; (**d**) V-I diagram of the output signals when ω = 7π; (**e**) V-I diagram of the output signals when ω = 10π; (**f **) V-I diagram of the output signals when ω = 12π.

From Fig. 9, it can be seen that when the number of loops remains constant, as the frequency of the driving voltage increases, the elliptical area of the V-I graph gradually increases, and the elliptical position also presents a state of gradually clockwise change with frequency change. This is completely consistent with the conclusion obtained from the simulation based on Laplace transform in Fig. 2.

When the circuit structure remains unchanged for forty loops and the driving voltage frequency changes, the current-time diagram of the output signal is as follows.

**Fig. 10:**
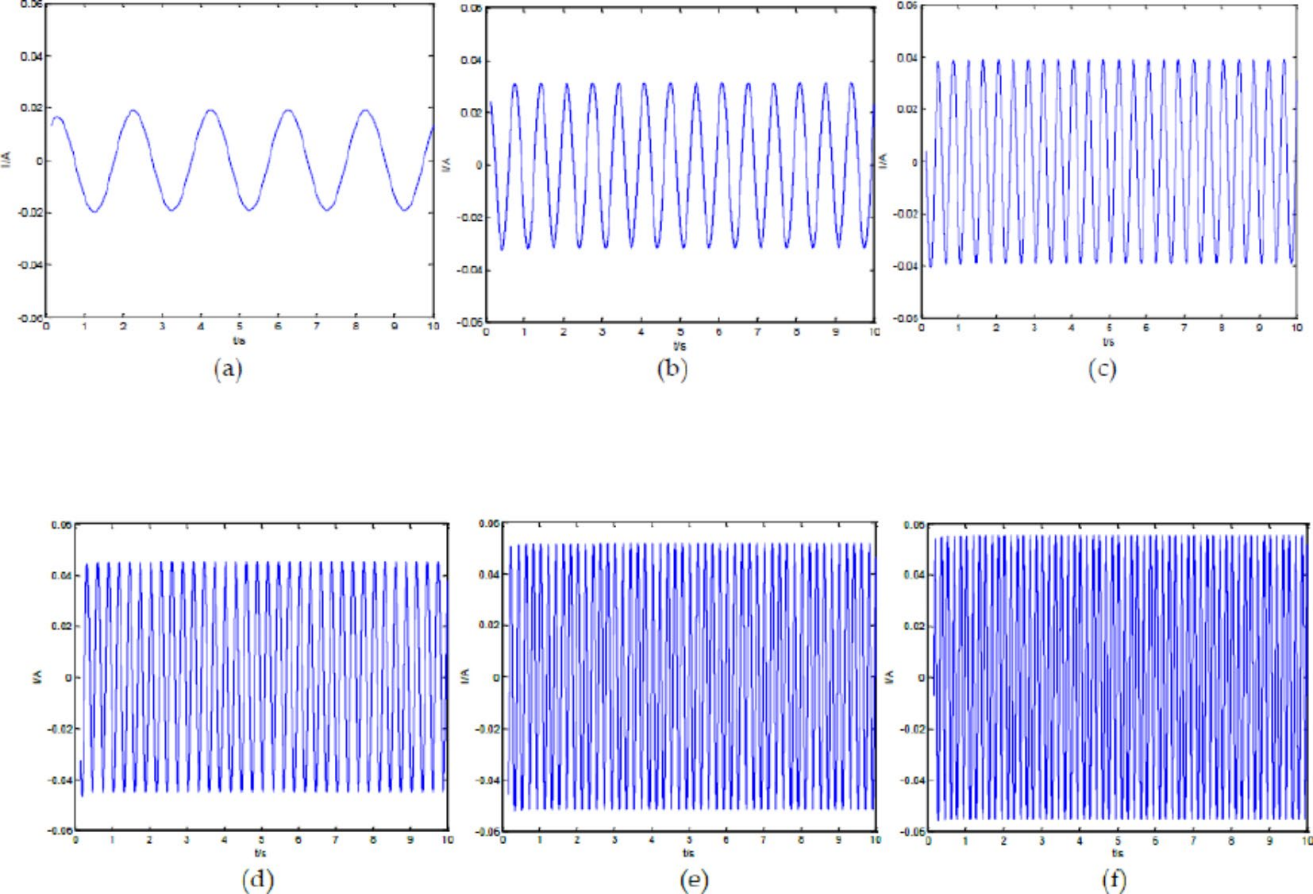
I-t diagram of Oldham chain fractance circuit with forty loops at different frequencies. (**a**) I-t diagram of the output signals when ω = 1π; (**b**) I-t diagram of the output signals when ω = 3π; (**c**) I-t diagram of the output signals when ω = 5π; (**d**) I-t diagram of the output signals when ω = 7π; (**e**) I-t diagram of the output signals when ω = 10π; (**f **) I-t diagram of the output signals when ω = 12π.

From Fig. 10, we can see that as the voltage frequency increases, the total current frequency and amplitude in the circuit also increase, which is completely consistent with the conclusion in Fig. 3.

Therefore, when using the LPIM to solve the Oldham fractal chain fractance circuit, the conclusions obtained are completely consistent with the ideal fractance properties obtained through operator approximation simulation based on Laplace transform and existing theories, which verifies the rationality and feasibility of the LPIM.

## Research on the output signals of FFMS

So far, most of the FMS theory is based on the net-grid-type structure circuit, and the memristors used are charge-controlled memristors. This chapter will construct a new FMS model and apply flux-controlled memristors to the circuit, resulting in FFMS.

### Construction and output study of Oldham fractal chain FMS

This article first applies memristors to Oldham fractal chain class structures. The structure of the 1/2-order Oldham fractal chain FMS circuit is shown in the following figure.

**Fig. 11:**
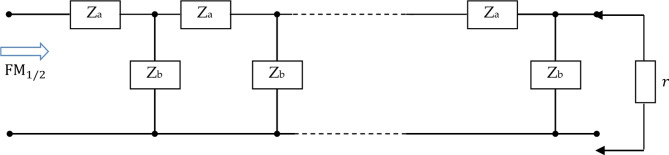
1 / 2 order Oldham chain FMS.

In Fig. 11, Z_a_ is the Laplace transform impedance of the memristor, and Z_b_ is the Laplace transform impedance of the classical passive capacitor or inductor. The circuit can be simplified and equivalent to the following circuits:

**Fig. 12:**
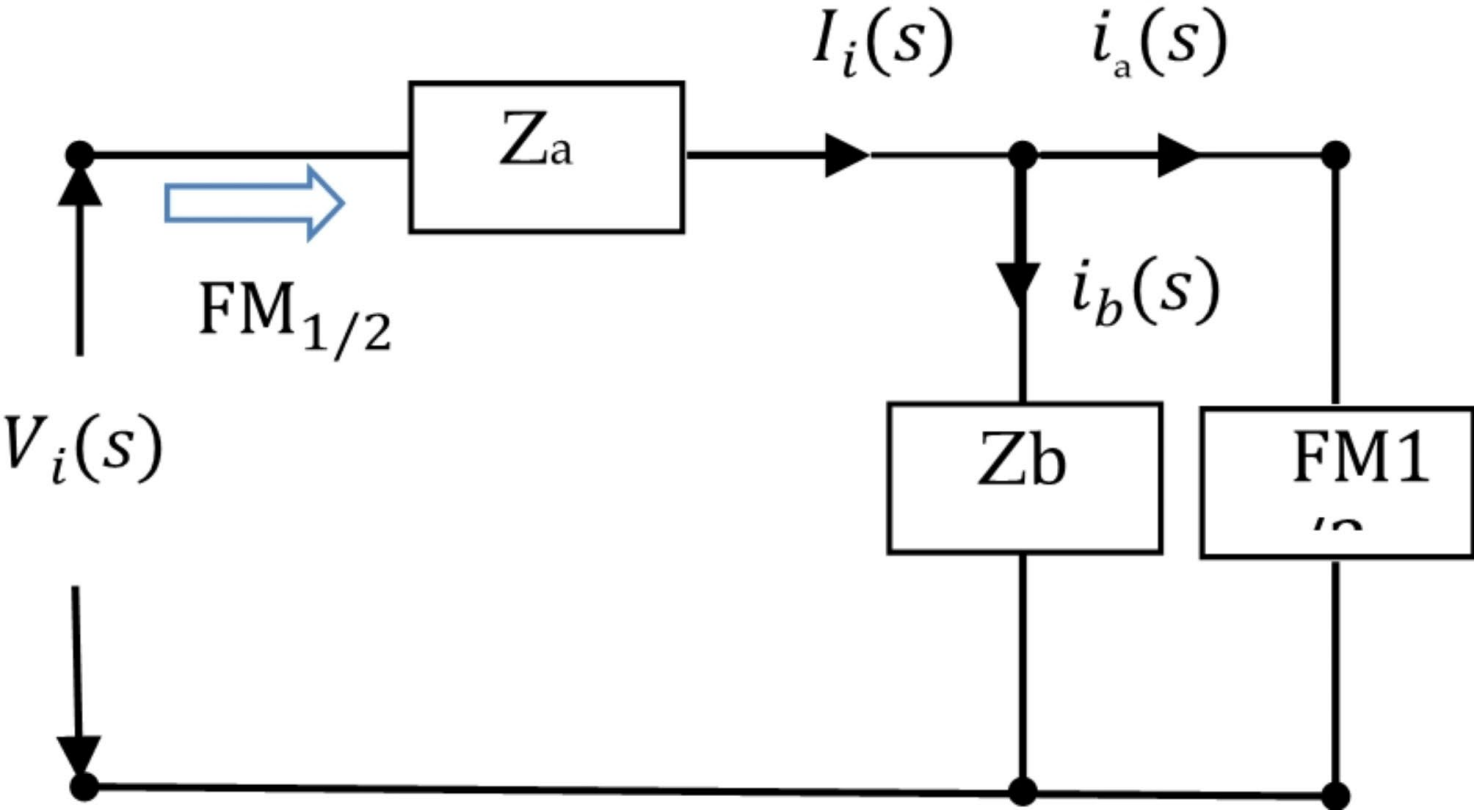
Simplified equivalent diagram of 1 / 2 order Oldham chain FMS.

As shown in Fig. 12, assuming the main circuit current passing through Z_a_ is $\:{I}_{i}\left(s\right)$, the two branch currents are${i_a}(s)$and ${i_b}(s)$, and the input voltage of the circuit is ${V_i}(s)$, according to the Kirchhoff voltage and current theorem, it can be obtained that:

25 $$\left\{ {\begin{array}{*{20}{l}} {{Z_a}{I_i}+{Z_b}{i_b}={V_i}} \\ {F{M_{{1 \mathord{\left/ {\vphantom {1 2}} \right. \kern-0pt} 2}}}({I_i} - {i_a}) - {Z_b}{i_b}=0} \end{array}} \right..$$

According to Eq. (25):

26 $${I_i}=\frac{{{Z_b}+F{M_{{1 \mathord{\left/ {\vphantom {1 2}} \right. \kern-0pt} 2}}}}}{{{Z_a}{Z_b}+({Z_a}+{Z_b})F{M_{{1 \mathord{\left/ {\vphantom {1 2}} \right. \kern-0pt} 2}}}}}{V_i}.$$

Then:

27 $$F{M_{{1 \mathord{\left/ {\vphantom {1 2}} \right. \kern-0pt} 2}}}=\frac{{{V_i}}}{{{I_i}}}=\frac{{{Z_a}{Z_b}+({Z_a}+{Z_b})F{M_{{1 \mathord{\left/ {\vphantom {1 2}} \right. \kern-0pt} 2}}}}}{{{Z_b}+F{M_{{1 \mathord{\left/ {\vphantom {1 2}} \right. \kern-0pt} 2}}}}}.$$

According to (27):

28 $$F{M_{{1 \mathord{\left/ {\vphantom {1 2}} \right. \kern-0pt} 2}}}=\frac{{{Z_a}+\sqrt {Z_{a}^{2}+4{Z_a}{Z_b}} }}{2}=\frac{{{Z_a}}}{2}\left( {1+\sqrt {1+\frac{{4{Z_b}}}{{{Z_a}}}} } \right).$$

Then $\:\frac{{Z}_{b}}{{Z}_{a}}\to\:\infty\:$, the above equation becomes:

29 $$F{M_{{1 \mathord{\left/ {\vphantom {1 2}} \right. \kern-0pt} 2}}} \approx \frac{{{Z_a}}}{2}\left( {\sqrt {\frac{{4{Z_b}}}{{Z{}_{a}}}} } \right)=\sqrt {{Z_a}{Z_b}} .$$

From this, it can be seen that this circuit approximates a 1/2-order FMS under certain conditions.

### Solving Oldham fractal chain capacitive FFMS using LPIM

Due to the special nature of memristors, it is extremely difficult to solve FMS circuits based on Laplace transform, and we have to use some non-universal memristors or approximations. Even so, so far, we are still unaware of the output signals of FFMS. This article proposes a new method for studying fractional order circuits—LPIM. Using this method, the output signals of FFMS will be simulated for the first time.

Taking Oldham fractal chain capacitive FMS as the research object, and using the flux-controlled memristor in this circuit, similarly, the circuit is first split. When there is only one loop in the circuit, the circuit structure is shown in the following figure.

**Fig. 13:**
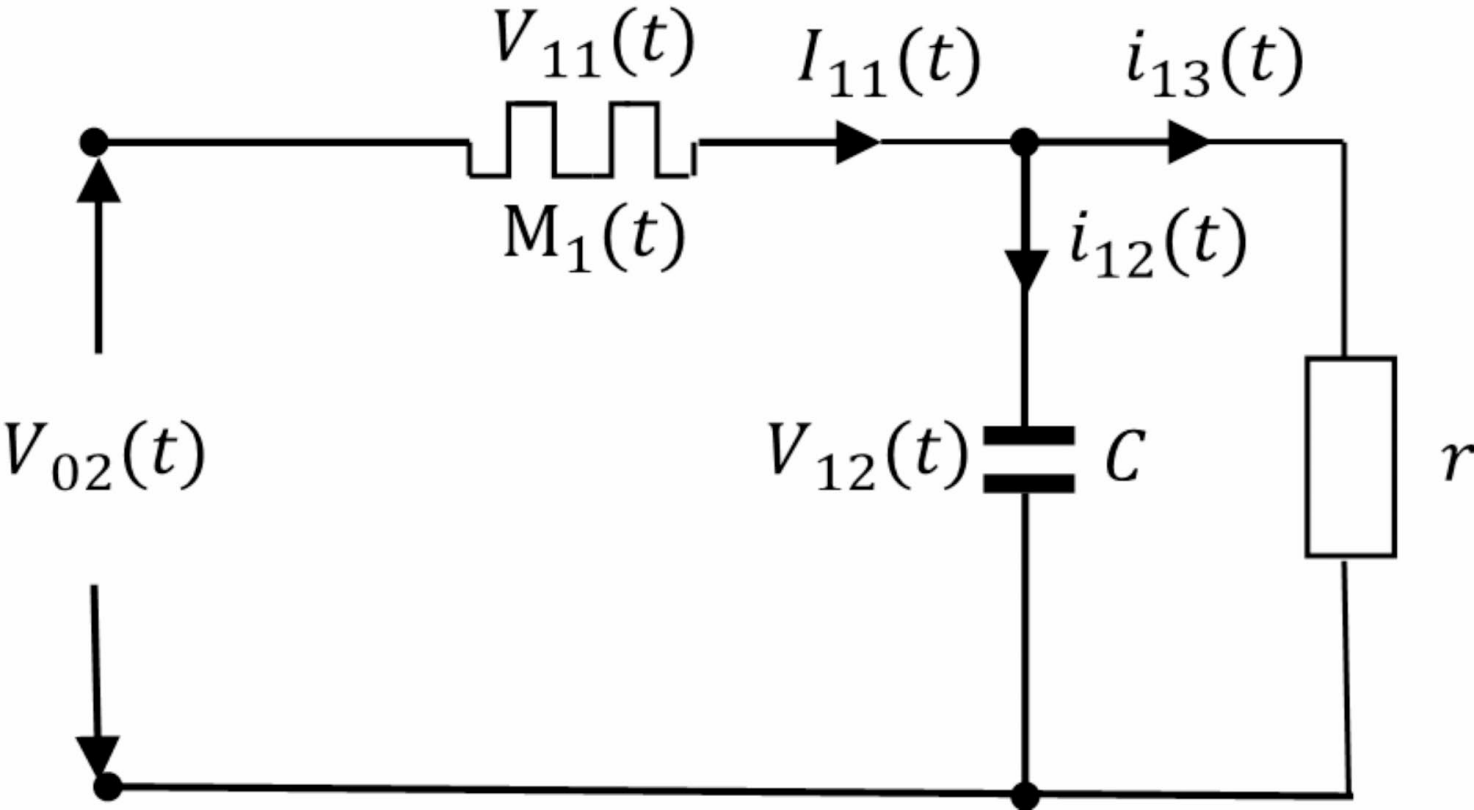
The Oldham chain FMS with only one loop.

In Fig. 13, where is the circuit symbol of memristor. The representation method of each physical quantity in the circuit is similar to that of fractance. According to Kirchhoff’s voltage and current theorem and the definition of capacitance, it is obtained that:

30 $$\left\{ {\begin{array}{*{20}{l}} {{V_{02}}={V_{11}}+{V_{12}}} \\ {{I_{11}}={i_{12}}+{i_{13}}} \\ {\frac{{{V_{11}}}}{{{M_1}}}={I_{11}}} \\ {\frac{{{V_{12}}}}{r}={i_{13}}} \\ {\frac{{d{V_{12}}}}{{dt}}=\frac{{{i_{12}}}}{C}} \end{array}} \right.$$

From Eq. (30), the final equation set is obtained:

31 $$\left\{ {\begin{array}{*{20}{l}} {\frac{{d{V_{12}}}}{{dt}}=\frac{{{V_{02}}}}{{C{M_1}}} - \left( {\frac{1}{{C{M_1}}}+\frac{1}{{Cr}}} \right){V_{12}}} \\ {\frac{{d{\varphi _1}}}{{dt}}={V_{11}}={V_{02}} - {V_{12}}} \end{array}} \right.,$$

where M_1_ is secondary nonlinear flux-controlled memristors, and ${M_1}=\frac{1}{{W{{(\varphi )}_1}}}=\frac{1}{{ - a+b\left| {{\varphi _1}} \right|}}$. This is a solvable system of first order differential equations.

When there are two loops in the circuit, the circuit structure is shown in the following figure.

**Fig. 14:**
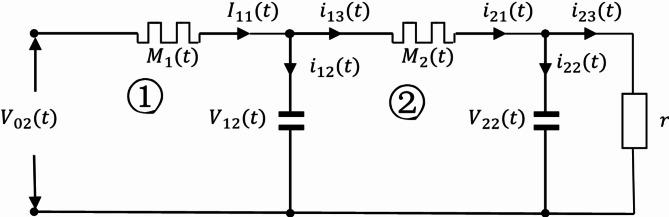
The Oldham chain FMS with two loops.

Similarly, from loop ① in Fig. 14, it can be concluded that:

32 $$\left\{ {\begin{array}{*{20}{l}} {{V_{02}}={V_{11}}+{V_{12}}} \\ {{I_{11}}={i_{12}}+{i_{13}}} \\ {\frac{{{V_{11}}}}{{{M_1}}}={I_{11}}} \\ {\frac{{d{V_{12}}}}{{dt}}=\frac{{{i_{12}}}}{C}} \\ {\frac{{d{\varphi _1}}}{{dt}}={V_{11}}} \end{array}} \right.,$$

where $${M_1}=\frac{1}{{W{{(\varphi )}_1}}}=\frac{1}{{ - a+b\left| {{\varphi _1}} \right|}}.$$

From loop ② in Fig. 14, it can be concluded that:

33 $$\left\{ {\begin{array}{*{20}{l}} {{V_{12}}={V_{21}}+{V_{22}}} \\ {{i_{21}}={i_{22}}+{i_{23}}} \\ {\frac{{{V_{21}}}}{{{M_2}}}={i_{21}}} \\ {\frac{{{V_{22}}}}{r}={i_{23}}} \\ {\frac{{d{V_{22}}}}{{dt}}=\frac{{{i_{22}}}}{C}} \end{array}} \right.,$$

where ${M_2}=\frac{1}{{W{{(\varphi )}_2}}}=\frac{1}{{ - a+b\left| {{\varphi _2}} \right|}}$. In Eqs. (32) and (33), ${i_{13}}={i_{21}}$, the following equation system is obtained through substitution:

34 $$\left\{ {\begin{array}{*{20}{l}} {\frac{{d{V_{12}}}}{{dt}}=\frac{{{V_{02}}{\text{-}}{V_{12}}}}{{C{M_1}}} - \frac{{{V_{12}} - {V_{22}}}}{{C{M_2}}}} \\ {\frac{{d{V_{22}}}}{{dt}}=\frac{{{V_{12}} - {V_{22}}}}{{C{M_2}}} - \frac{{{V_{22}}}}{{Cr}}} \\ {\frac{{d{\varphi _1}}}{{dt}}={V_{11}}=V - {V_{12}}} \\ {\frac{{d{\varphi _2}}}{{dt}}={V_{21}}={V_{12}} - {V_{22}}} \end{array}} \right.,$$

where ${M_1}=\frac{1}{{W{{(\varphi )}_1}}}=\frac{1}{{ - a+b\left| {{\varphi _1}} \right|}}$, ${M_2}=\frac{1}{{W{{(\varphi )}_2}}}=\frac{1}{{ - a+b\left| {{\varphi _2}} \right|}}$. Equation (34) is also a solvable system of differential equations.

When the number of circuit loops increases to three, the circuit structure is shown in the following figure.

**Fig. 15:**
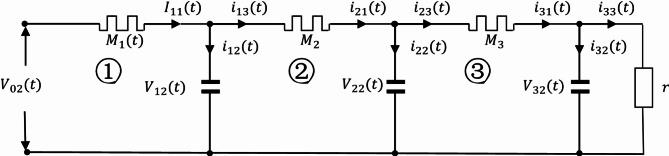
The Oldham chain FMS with three loops.

Similarly, from loop ① in Fig. 15, it can be concluded that:

35 $$\left\{ {\begin{array}{*{20}{l}} {{V_{02}}={V_{11}}+{V_{12}}} \\ {{I_{11}}={i_{12}}+{i_{13}}} \\ {\frac{{{V_{11}}}}{{{M_1}}}={I_{11}}} \\ {\frac{{d{V_{12}}}}{{dt}}=\frac{{{i_{12}}}}{C}} \\ {\frac{{d{\varphi _1}}}{{dt}}={V_{11}}} \end{array}} \right.,$$

where $${M_1}=\frac{1}{{W{{(\varphi )}_1}}}=\frac{1}{{ - a+b\left| {{\varphi _1}} \right|}}$$

From loop ② in Fig. 15, it can be concluded that:

36 $$\left\{ {\begin{array}{*{20}{l}} {{V_{12}}={V_{21}}+{V_{22}}} \\ {{i_{21}}={i_{22}}+{i_{23}}} \\ {\frac{{{V_{21}}}}{{{M_2}}}={i_{21}}} \\ {\frac{{d{V_{22}}}}{{dt}}=\frac{{{i_{22}}}}{C}} \\ {\frac{{d{\varphi _2}}}{{dt}}={V_{21}}} \end{array}} \right.,$$

where $${M_2}=\frac{1}{{W{{(\varphi )}_2}}}=\frac{1}{{ - a+b\left| {{\varphi _2}} \right|}}.$$

From loop ③ in Fig. 15, it can be concluded that:

37 $$\left\{ {\begin{array}{*{20}{l}} {{V_{13}}={V_{31}}+{V_{32}}} \\ {{i_{31}}={i_{32}}+{i_{33}}} \\ {\frac{{{V_{31}}}}{{{M_3}}}={i_{31}}} \\ {\frac{{{V_{32}}}}{r}={i_{33}}} \\ {\frac{{d{V_{32}}}}{{dt}}=\frac{{{i_{32}}}}{C}} \end{array}} \right.,$$

where $${M_3}=\frac{1}{{W{{(\varphi )}_3}}}=\frac{1}{{ - a+b\left| {{\varphi _3}} \right|}}.$$

Because ${i_{13}}={i_{21}}$ and ${i_{23}}={i_{31}}$, it can be obtained from (35), (36), and (37)

38 $$\left\{ {\begin{array}{*{20}{l}} {\frac{{d{V_{12}}}}{{dt}}=\frac{{{V_{02}}{\text{-}}{V_{12}}}}{{C{M_1}}} - \frac{{{V_{12}} - {V_{22}}}}{{C{M_2}}}} \\ {\frac{{d{V_{22}}}}{{dt}}=\frac{{{V_{12}} - {V_{22}}}}{{C{M_2}}} - \frac{{{V_{22}}}}{{C{M_3}}}} \\ {\frac{{d{V_{32}}}}{{dt}}=\frac{{{V_{22}} - {V_{32}}}}{{C{M_3}}} - \frac{{{V_{32}}}}{{Cr}}} \\ {\frac{{d{\varphi _1}}}{{dt}}={V_{11}}=V - {V_{12}}} \\ {\frac{{d{\varphi _2}}}{{dt}}={V_{21}}={V_{12}} - {V_{22}}} \\ {\frac{{d{\varphi _3}}}{{dt}}={V_{31}}={V_{22}} - {V_{32}}} \end{array}} \right.,$$

where ${M_1}=\frac{1}{{W{{(\varphi )}_1}}}=\frac{1}{{ - a+b\left| {{\varphi _1}} \right|}}$, ${M_2}=\frac{1}{{W{{(\varphi )}_2}}}=\frac{1}{{ - a+b\left| {{\varphi _2}} \right|}}$, ${M_3}=\frac{1}{{W{{(\varphi )}_3}}}=\frac{1}{{ - a+b\left| {{\varphi _3}} \right|}}$. Equation (38) is still a solvable system of first order differential equations.

By analogy, when the number of circuit loops is n, the circuit structure is shown in the following figure.

**Fig. 16:**
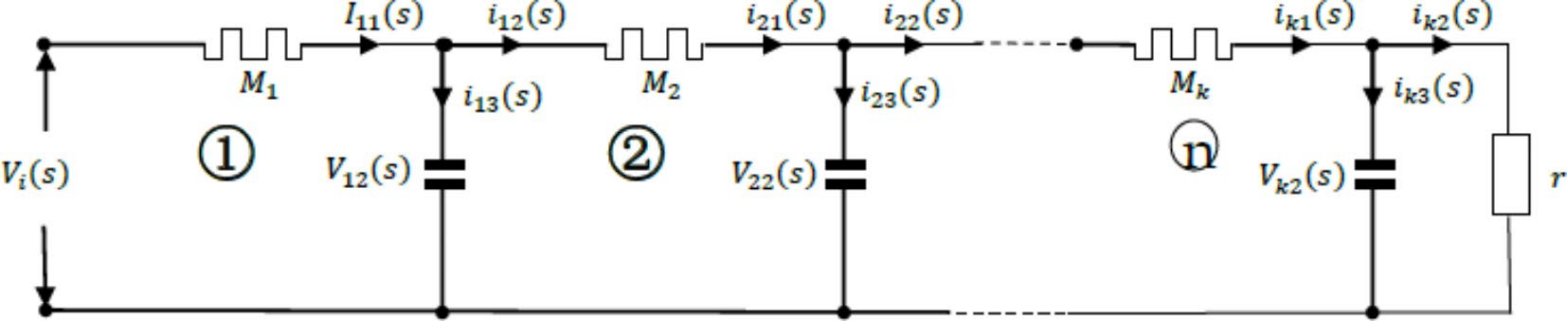
The Oldham chain FMS with n loops.

Figure 16 shows the Oldham chain FMS structure with n loops, and its differential equation system is:

39 $$\left\{ \begin{aligned}&\frac{d{{V}_{k2}}}{dt}=\frac{{{V}_{(k-1)2}}\text{-}{{V}_{k2}}}{C{{M}_{k}}}-\frac{{{V}_{k2}}-{{V}_{(k+1)2}}}{C{{M}_{(k+1)}}}\ \quad k=1,2,3,\ldots ,n-1 \\&\frac{d{{V}_{k2}}}{dt}=\frac{{{V}_{(k-1)2}}\text{-}{{V}_{k2}}}{C{{M}_{(k-1)}}}-\frac{{{V}_{k2}}}{Cr}\quad \quad \quad\qquad \,\, k=n \\& \frac{d{{\varphi }_{k}}}{dt}={{V}_{k1}}={{V}_{(k-1)2}}-{{V}_{k2}}\quad \quad \qquad \quad k=1,2,3,\ldots,n \\\end{aligned} \right.$$

where $${M_k}=\frac{1}{{W{{(\varphi )}_k}}}=\frac{1}{{ - a+b\left| {{\varphi _k}} \right|}}$$$$k=1,2,3, \ldots ,n$$. By using MATLAB, the differential equation system (39) can be solved and the output signals of FFMS can be simulated.

Make the input voltage $\:{\text{V}}_{1}={V}_{max}\text{s}\text{i}\text{n}\left(\omega\:t\right)$, ${V_{max}}=3\,V$, $\:\omega\:=3\pi\:$; $\:\:a=0.7$, $\:b=1.48$, $\:r=2000{\Omega\:}$, $C=1\,F$. V-I diagrams of the FFMS output with different circuit loop numbers:

**Fig. 17:**
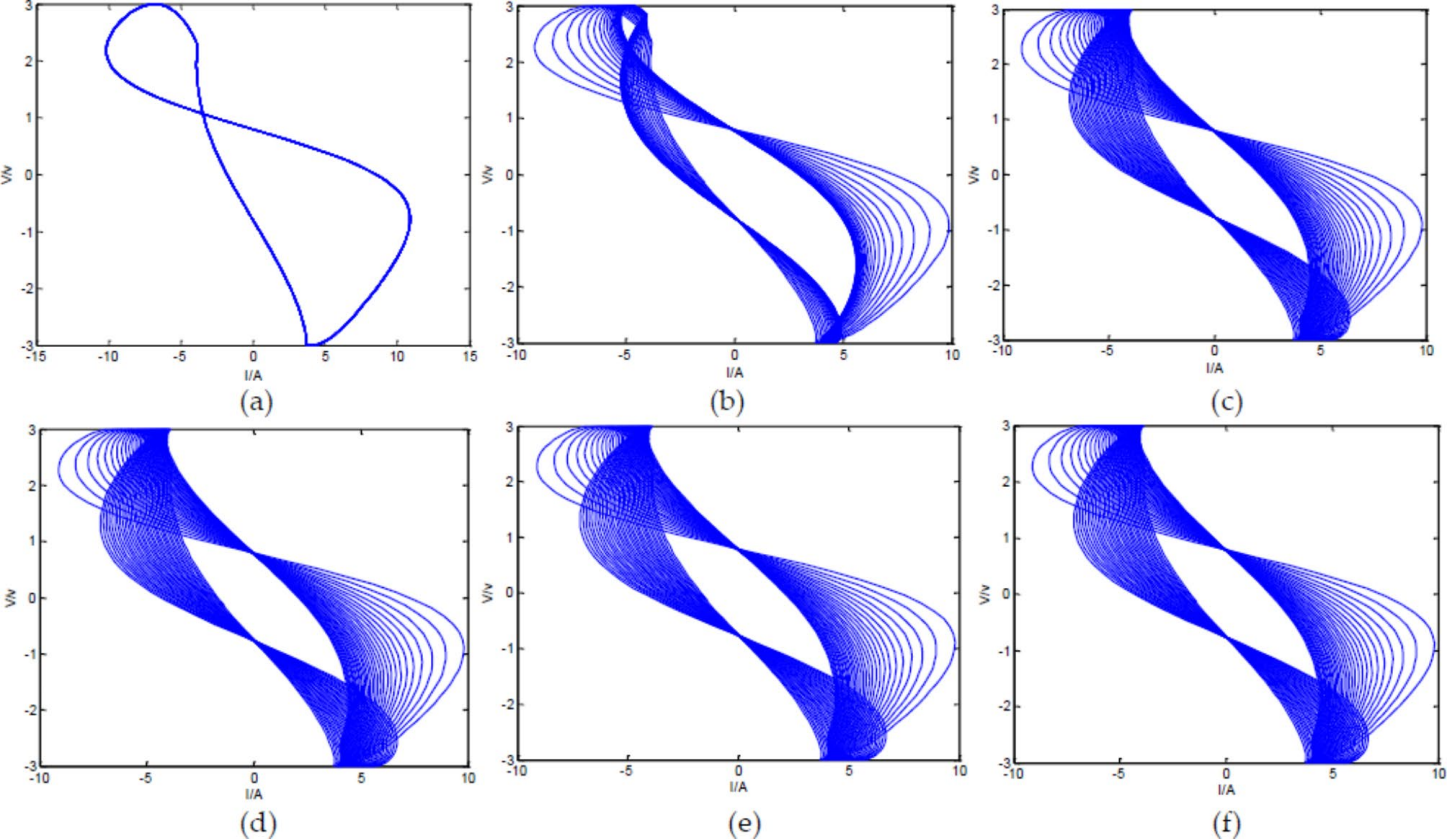
V-I diagram of Oldham chain FFMS with different loop numbers. (**a**) V-I diagram of the output signals with three loops; (**b**) V-I diagram of the output signals with five loops; (**c**) V-I diagram of the output signals with ten loops; (**d**) V-I diagram of the output signals with twenty loops; (**e**) V-I diagram of the output signals with thirty loops; (**f**) V-I diagram of the output signals with forty loops.

From Fig. 17, it can be seen that when the circuit has only three loops, the system does not exhibit the characteristic of FMS, but rather exhibits a characteristic similar to memristor. When the number of loops in the circuit increases to 5, the system basically has the output property of FMS. However, when the number of loops is ten, twenty, thirty, and forty, the output of the circuit is almost the same, indicating that the properties of the circuit are relatively stable and approach the ideal FMS when the number of loops is about ten.

The V-I diagram of the FFMS output signals intersects at two points, where the voltage is symmetrical about the origin and the current is equal, which is different from both fractance and memristor. This is similar to the conclusion obtained in the net-grid-type FMS in reference^50^. Different circuit structures: one is net-grid-type circuit structure, and the other is Oldham fractal chain structure; Different types of memristors: one used charge-controlled memristor, and the other used secondary nonlinear flux-controlled memristor; Different processing methods: one used Laplace transform, and the other used LPIM; But the same conclusion was reached: the V-I graph intersects at two points. Therefore, it is highly likely that the V-I diagrams of FMS intersect at two points.

When the number of circuit loops remains unchanged at forty, while the frequency of the driving voltage changes and other parameters remain unchanged, the V-I diagram of the FFMS output is shown in the following figure.

**Fig. 18:**
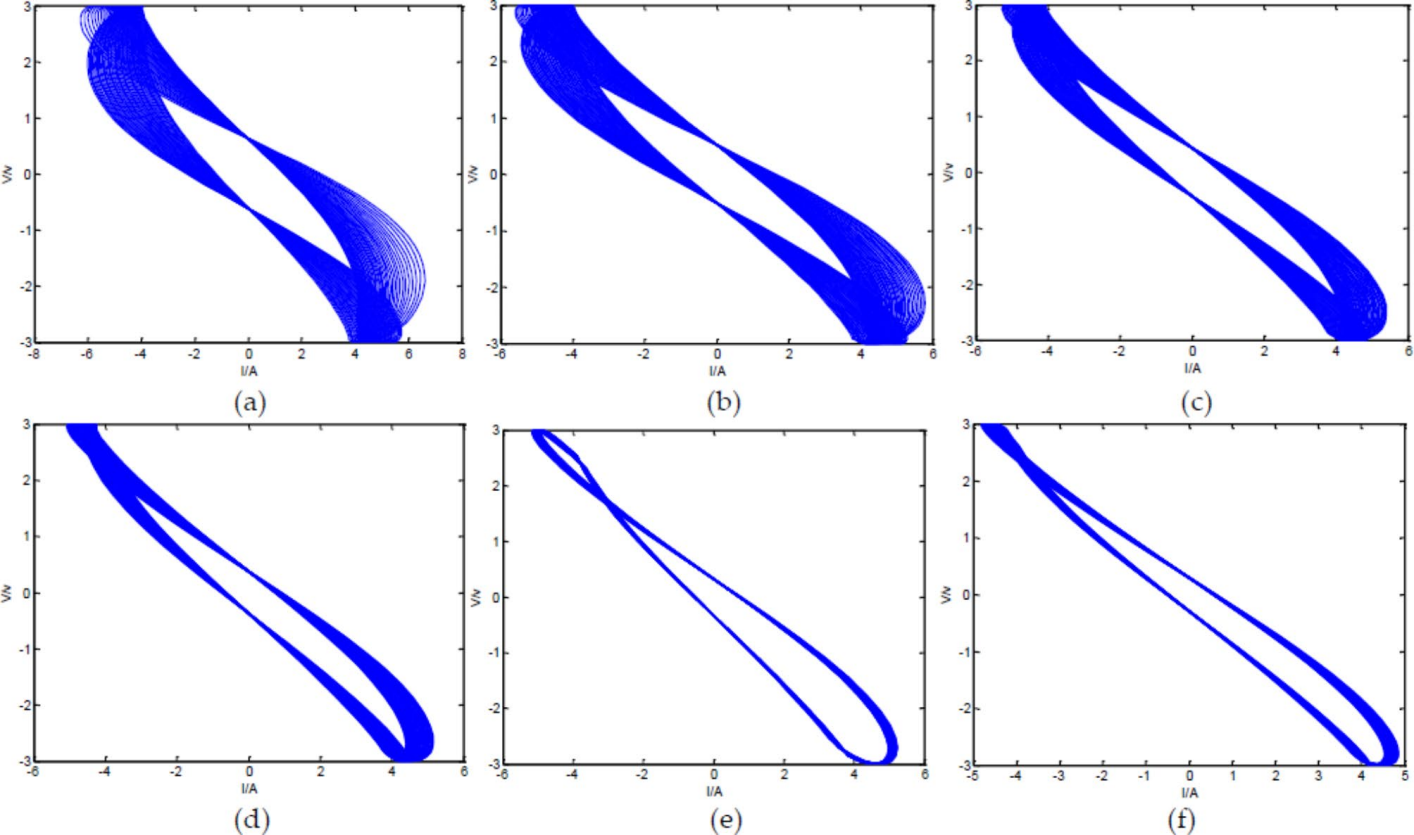
V-I diagram of Oldham chain FFMS with forty loops at different frequencies. (**a**) V-I diagram of the output signals when $\:{\upomega\:}=4{\uppi\:}$; (**b**) V-I diagram of the output signals when $\:{\upomega\:}=5{\uppi\:}$; (**c**) V-I diagram of the output signals when $\:{\upomega\:}=6{\uppi\:}$; (**d**) V-I diagram of the output signals when $\:{\upomega\:}=7{\uppi\:}$; (**e**) V-I diagram of the output signals when $\:{\upomega\:}=8{\uppi\:}$; (**f**) V-I diagram of the output signals when $\:{\upomega\:}=9{\uppi\:}$

From Fig. 18, it can be seen that as the frequency increases, the V-I diagram of the output signals gradually changes. When the frequency increases to a certain value, the circuit exhibits characteristics similar to memristor, that is, the shape of V-I diagram is similar to Fig. 8. This is mainly because when the frequency increases to a certain value, the capacitance in the circuit is equivalent to a short circuit state, and the circuit becomes a memristor connection.

From Figs. 17 and 18, it can be seen that compared to fractances and memristors, the dynamic behavior of FMS is more complex. This is because the memristor in FMS is a non-volatile nonlinear component, and the FMS constructed using memristors and capacitors has electrical characteristics between capacitors and memristors, making the dynamic behavior of FMS more complex. Due to the inherent nonlinearity of memristors, they have been widely used in chaotic oscillation circuits, and the dynamic behavior of FMS is more complex and sensitive to parameter changes, thus having great potential for application in chaotic oscillation circuits. However, due to the complex solving problems, the application of FMS in chaotic oscillation circuits is just beginning^51–55^. The proposal of LPIM hopes to better promote the research of FMS application in chaotic oscillation circuits.

## Conclusion

A new research method for fractional-order circuits, namely LPIM, has been proposed. This method can solve any structure of FMS constructed by any form of memristor, which effectively solves the problem of solving FMS. To demonstrate the rationality of LPIM, fractance circuits with relatively simple forms in fractional-order circuits were taken as the research object. The output signals of Oldham fractal chain fractance circuit were studied using LPIM and traditional Laplace transform, respectively. The results showed that the conclusions obtained by LPIM were completely consistent with those obtained by Laplace transform and existing theories. Then, for the first time, this article applies memristors to Oldham fractal chain circuits and constructs a new type of FMS. Through proof, it can be seen that this is a non-ideal FMS. Then, the LPIM is used to solve the properties of the FFMS under this structure, which is the first simulation to obtain the output signals of the FFMS. From the simulation results, it can be seen that when other parameters in the circuit remain unchanged and the number of loops in the circuit changes, the characteristics of the output signals become more stable as the number of loops increases. Its V-I diagram intersects at two points, where the voltage is symmetrical about the origin and the current is equal. This is similar to the conclusions obtained in existing theories, so we boldly speculate that the V-I diagram of FMS output signals is likely to intersect at two points. When the frequency changes and other parameters remain unchanged, the V-I diagram gradually changes as the frequency increases. When the frequency increases to a certain value, the output exhibits characteristics similar to memristor, that is, the shape of V-I diagram is similar to the shape of 8. This is mainly because when the frequency increases to a certain value, the capacitors in the circuit are equivalent to a short circuit state, and the circuit becomes a memristor connection. From the output signal of FMS, it can also be seen that the dynamic behavior of FMS is more complex, which is due to its electrical properties being between capacitance and nonlinear element memristor. This makes it have great potential for application in chaotic systems.

## Data Availability

All data included in this study are available upon request by contact with the corresponding author.
